# The long-term results and prognostic significance of cutaneous melanoma surgery using sentinel node biopsy with triple technique

**DOI:** 10.1186/s12957-015-0701-8

**Published:** 2015-10-13

**Authors:** Piotr Rutkowski, Konrad Szydłowski, Zbigniew I. Nowecki, Maciej Sałamacha, Tomasz Goryń, Beata Mitręga-Korab, Andrzej Pieńkowski, Wirginiusz Dziewirski, Marcin Zdzienicki

**Affiliations:** Department of Soft Tissue/Bone Sarcoma and Melanoma, Maria Sklodowska-Curie Memorial Cancer Center and Institute of Oncology, Roentgena 5, 02-781 Warsaw, Poland; Department of General Surgery, Elblag, Poland

**Keywords:** Melanoma, Sentinel lymph node biopsy, Mitotic rate, Non-sentinel lymph node, Prognosis, Tumor burden

## Abstract

**Background:**

The sentinel lymph node biopsy (SLN) is a basic staging method in all primary cutaneous melanomas ≥pT1b. The standard technique is a triple technique consisting of preoperative lymphoscintigraphy, intraoperative blue-dye lymphography, and gamma-probe assessment. We performed the analysis of long-term results in a very large one-institution series of cutaneous melanoma patients.

**Methods:**

We have analyzed treatment results of a group of 1764 consecutive patients with cutaneous melanoma, who underwent SLN biopsy between 1997 and 2008 in one tertiary center. Additionally, we have analyzed the outcomes of a group of 473 patients with positive SLN biopsy undergoing completion lymph node dissection (CLND). Median follow-up time was 5.3 years.

**Results:**

Metastases to SLN (SLN+) were found in 19.9 %. Eight-year overall survival (OS) rate in the entire group was 73.5 %, 80 % without SLN metastases (SLN−) and 50 % in group with SLN+ (*p* < 0.001). Independent prognostic factors for OS were as follows: presence of metastases to SLN, primary tumor ulceration, and higher mitotic index (>5/mm^2^) of primary tumor. The nodal recurrences in the biopsied lymphatic basin were 5.4 %. The metastases to non-sentinel lymph nodes (NSLN found in 27 % of patients with SLN+) correlated (on multivariable logistic regression analysis) with primary tumor thickness >4 mm, SLN metastatic deposit size >1 mm, and extracapsular involvement of SLN. In an additionally analyzed SLN+ group, the NSLN involvement was related to poorer prognosis (8-year OS rate NSLN− vs NSLN+: 59.6 vs. 34.7 %, respectively). The independent prognostic factors for OS in the SLN+ group were a higher Breslow thickness and ulceration of primary tumor, metastases to more than 1 lymph nodes.

**Conclusions:**

The long-term results confirm crucial prognostic significance of SLN biopsy in cutaneous melanoma. We identified factors related to NSLN involvement, which in the future may limit indications for CLND.

## Background

Several studies have already proven that sentinel lymph node (SLN) biopsy offers several benefits in the course of melanoma patient management: excellent prognostic information, better staging, avoiding unnecessary elective lymph node dissection (LND), facilitation of therapeutic lymphadenectomy, homogeneity of patient populations in clinical trials on adjuvant therapy, patients’ increased sense of safety, and accuracy of care [1–4].

SLN biopsy developed in the early 1990s [5], in 1999 was declared by the World Health Organization as a standard of care in the management of melanoma patients without evidence of metastases, and thereafter the American Joint Committee on Cancer (AJCC) incorporated SLN biopsy as a microstaging procedure in the TNM-staging system [6, 7]. In 2012, the American Society of Surgical Oncology and Society of Surgical Oncology confirmed that SLN biopsy is recommended in all primary melanoma patients with a Breslow thickness >1 mm and also in those patients with thinner melanomas but at the same time with other negative pathological features. Finally, the definitive analysis from the Multicenter Selective Lymphadenectomy Trial-1 (MSLT-1), which randomized patients into those who underwent SLN biopsy and others who did not, was published in 2014 [8].

Currently, we can present the long-term outcomes of SLN biopsy used in routine practice based on a very large one-institution series of cutaneous melanoma patients, and we can focus on some issues reflecting the relationship between pathological characteristics of the tumor and SLN with patients’ survival.

We performed the analysis of long-term results of SLN biopsy, and additionally, in the subgroup of patients, we analyzed the impact of new possible prognostic factors on patient outcomes, including mitotic index of the primary tumor (introduced to the AJCC staging system in 2009) and features of SLN metastases.

## Methods

### Patients

We analyzed the outcomes of 1764 consecutive patients with histologically confirmed primary cutaneous melanoma in clinical stages I–II according to the AJCC 2010 classification [9], who underwent sentinel node biopsy at the Department of Soft Tissue/Bone Sarcoma and Melanoma at the Maria Sklodowska-Curie Memorial Cancer Centre and Institute of Oncology, Warsaw, Poland (CCIO), between 1998 and 2008 (cohort 1) (Table 1). In all patients, the triple technique was used consisting of preoperative lymphoscintigraphy, blue-dye injection, and intraoperative lymphoscintigraphy with a hand-held gamma-detecting probe. We have already presented the detailed technique of SNB and of the pathologic examination of SLNs in our previous publications [1]. The false-negative cases were defined as a nodal recurrence after an initially negative SLN biopsy in the biopsied basin without preceding local or in-transit recurrences. In the case of positive SLN, all but three patients underwent completion lymph node dissection (CLND). The margin of excision of all the primaries was ≥1 cm. Each patient provided written informed consent. The study was approved by the local Bio-Ethics Committee according to Good Clinical Practice Guidelines.

**Table 1:** Patient characteristics of all patients undergoing SLN biopsy (cohort 1) and overall survival (OS)

Parameter	Value	*N* (%)	5-year OS	95 % CI	8-year OS	95 % CI	*p* value log-rank test
Płeć	Female	1021 (57.9 %)	84.8	(82.2–87.4)	78.6	(74.9–82.5)	0.001
	Male	743 (42.1 %)	72.3	(68.5–76.4)	66.6	(61.6–72.0)	
Tumor type	NM	695 (39.4 %)	74.0	(70.2–78.0)	67.5	(62.8–72.5)	0.0001
	SSM	516 (29.25 %)	87.1	(83.7–90.6)	82.2	(77.0–87.9)	
	ALM	44 (2.49 %)	65.0	(51.0–82.8)	65.0	(51.0–82.8)	
	LMM	122 (6.92 %)	84.3	(76.6–92.7)	77.5	(66.7–90.1)	
	Other	9 (0.51 %)	83.3	(58.3–100.0)	83.3	(58.3–100.0)	
	NA	378 (21.43 %)	80.0	(75.0–85.4)	73.2	(66.1–81.0)	
Clark level	2	253 (14.34 %)	90.7	(86.3–95.3)	84.2	(77.0–92.2)	0.001
	3	818 (46.37 %)	86.1	(83.3–89.0)	78.8	(74.5–83.4)	
	4	490 (27.78 %)	68.7	(63.8–73.9)	65.4	(60.1–71.3)	
	5	110 (6.24 %)	54.7	(44.9–66.6)	47.2	(36.2–61.5)	
	NA	93 (5.27 %)	78.9	(69.7–89.3)	78.9	(69.7–89.3)	
Ulceration of primary tumor	0	931 (52.78 %)	90.4	(88.1–92.7)	84.8	(81.0–88.8)	0.001
	1	713 (40.59 %)	65.7	(61.7–70.0)	58.9	(54.1–64.3)	
	NA	115 (6.63 %)	79.5	(70.9–89.1)	76.5	(66.8–87.7)	
Extracapsular involvement of sentinel node metastases	0	321	62.4	(56.7–68.7)	54.2	(47.5–61.7)	0
	1	96	33.8	(25.1–45.6)	27.0	(18.6–39.2)	
	NA	1341	88.6	(86.4–90.7)	84.2	(81.0–87.4)	
Mitotic index	<1/mm^2^	123 (15.28 %)	92.9	(86.0–100.0)	92.9	(86.0–100.0)	0.002
	1/mm^2^	142 (17.64 %)	89.9	(81.2–99.5)	89.9	(81.2–99.5)	
	2–5/mm^2^	262 (32.55 %)	88.3	(83.3–93.6)	84.7	(78.0–92.0)	
	>5/mm^2^	278 (34.53 %)	63.1	(56.4–70.7)	61.6	(54.5–69.7)	
Breslow thickness	≤1 mm	343 (19.44 %)	94.2	(91.0–97.4)	88.7	(82.7–95.2)	0.001
	1.01–2.00 mm	449 (25.45 %)	91.5	(88.5–94.7)	87.8	(83.1–92.8)	
	2.01–4.00 mm	519 (29.42 %)	75.6	(71.1–80.3)	68.0	(61.8–74.7)	
	>4 mm	398 (22.56 %)	60.8	(55.5–66.6)	54.4	(48.4–61.0)	
	NA	55 (3.12 %)	75.4	(62.8–90.4)	70.9	(57.1–88.1)	
Sentinel node metastases	No	1413 (80.10 %)	86.3	(84.1–88.5)	80.4	(77.1–83.9)	0.001
	Yes	351 (19.9 %)	55.6	(50.1–61.9)	49.8	(43.7–56.8)	
All patients		1764	79.5	(77.2–81.8)	73.5	(70.5–76.7)	

All patients undergoing SLN biopsy met the following criteria:

- Primary focus cutaneous melanoma after excisional biopsy with Breslow thickness ≥0.75 mm or ulcerated or Clark level ≥IV (all histological diagnoses were confirmed in the Department of Pathology, CCIO)
- Clinically non-palpable regional lymph nodes
- Absence of distant metastases (confirmed routinely by physical examination, chest X-ray, and ultrasonography of the abdominal cavity)
- Feasibility for general anesthesia

The patients had not undergone any other preliminary selection. Only patients who met with all the conditions listed above were enrolled in the study.

The clinico-pathological stage of the melanoma patients was determined by pathological evaluation of the primary lesion and of the dissected lymph nodes, as well as by physical examination and routine imaging examinations (chest X-ray, ultrasonography of the abdominal cavity, and computed tomography imaging, if necessary).

Patient characteristics of the cohort 1 are summarized in Table 1. In an additional 805 cases, two pathologists reviewed mitotic index per mm^2^. All patients had confirmed primary melanoma. Radical lymph node dissections were performed according to the technique described by Karakoussis [10]. For ilio-inguinal lymphadenectomy, we routinely excised the superficial and deep levels below the inguinal ligament to the level of the aortic bifurcation combined with obturatory lymph node dissection. Two hundred and one patients received interferon-α2b as adjuvant treatment in accordance with the European Organisation for Research and Treatment of Cancer (EORTC) 18952 trial (without significant influence on overall survival data) [11, 12].

Additionally, we analyzed all consecutive patients (*N* = 473) who underwent radical CLND at the Department of Soft Tissue/Bone Sarcoma and Melanoma at the CCIO between May 1995 and December 2008 due to positive SLN biopsy (cohort 2) independent of the SLN biopsy technique used (Table 2).

**Table 2:** Patient characteristics of patients undergoing completion lymph node dissection due to positive SLN biopsy (cohort 2) and overall survival (OS) *N* = 473

Parameter	Value	*N* (%)	5-year OS	95 % CI	8-year OS	95 % CI	*p* value log-rank test
Sex	Female	235 (49.6 %)	63.0	(56.4–70.5)	59.1	(52.1–67.0)	0.032
	Male	238 (50.4 %)	51.2	(44.2–59.4)	44.8	(37.3–53.9)	
Tumor type	NM	264	57.2	(50.6–64.6)	51.0	(44.1–59.1)	0.006
	SSM	96	63.8	(53.7–75.8)	61.1	(50.5–74.1)	
	ALM	15	20.0	(6.3–63.8)	20.0	(6.3–63.8)	
	LMM	16	46.1	(25.2–84.3)	46.1	(25.2–84.3)	
	Other	2	100.0	(100.0–100.0)	100.0	(100.0–100.0)	
	NA	80	58.7	(47.4–72.7)	54.0	(42.3–68.9)	
Clark level	II	29	49.4	(31.7–77.1)	43.2	(25.8–72.4)	0.068
	III	154	69.8	(62.2–78.4)	61.4	(52.1–72.4)	
	IV	206	51.9	(44.2–60.9)	50.8	(43.1–59.9)	
	V	64	46.3	(34.8–61.5)	39.6	(28.3–55.3)	
	NA	20	63.6	(43.8–92.3)	56.5	(36.5–87.6)	
Presence of ulceration of primary tumor	No	143	73.2	(65.0–82.3)	68.4	(59.5–78.7)	0.000
	Yes	293	49.0	(42.7–56.1)	43.8	(37.3–51.4)	
	NA	37	60.9	(45.7–81.4)	57.1	(41.7–78.3)	
Extracapsular involvement of sentinel node metastases	No	363	58.5	(53.0–64.7)	52.7	(46.8–59.4)	0.008
	Yes	98	40.1	(27.8–57.8)	37.2	(25.1–55.2)	
	NA	12	68.9	(54.4–87.4)	68.9	(54.4–87.4)	
Breslow thickness	≤1 mm	29	81.9	(67.3–99.8)	70.6	(53.0–94.0)	0.000
	1.01–2.00 mm	56	75.6	(63.8–89.5)	75.6	(63.8–89.5)	
	2.01–4.00 mm	164	61.9	(53.4–71.8)	57.6	(48.1–68.9)	
	>4 mm	199	44.3	(37.2–52.8)	39.8	(32.6–48.6)	
	NA	25	60.8	(40.8–90.5)	43.4	(23.5–80.3)	
Size of metastasis in sentinel node	<0.1 mm	10	87.5	(67.3–100.0)	87.5	(67.3–100.0)	0.009
	0.1–1.0 mm	107	63.0	(53.6–74.2)	57.7	(47.0–71.0)	
	>10.0 mm	246	47.3	(40.3–55.6)	41.6	(34.4–50.4)	
	NA	110	56.8	(45.3–71.2)	48.4	(36.2–64.7)	
Localization of metastasis in sentinel node	Subcapsular	25	79.4	(63.1–100.0)	79.4	(63.1–100.0)	0.060
	Combined	165	57.8	(49.7–67.1)	51.5	(42.4–62.5)	
	Parenchymal	67	52.2	(40.6–67.2)	44.0	(32.0–60.6)	
	Multifocal	15	55.0	(32.2–93.8)	55.0	(32.2–93.8)	
	Extensive	75	40.8	(29.8–55.9)	38.5	(27.6–53.8)	
	NA	126	66.8	(57.5–77.6)	61.7	(51.8–73.5)	
Presence of non-sentinel node metastases	No	341	63.9	(58.2–70.2)	59.6	(53.5–66.3)	0.000
	Yes	132	41.4	(32.9–52.2)	34.7	(25.9–46.6)	
LND basin	Axillary	261	56.4	(49.8–64.0)	49.5	(42.1–58.1)	0.316
	Neck	3	50.0	(12.5–100.0)	50.0	(12.5–100.0)	
	Inguinal	174	63.0	(55.4–71.5)	59.0	(51.2–68.1)	
	Other	35	28.9	(14.3–58.6)	28.9	(14.3–58.6)	
Number of metastatic nodes	1	259	66.3	(60.0–73.3)	62.7	(55.9–70.3)	0.000
	2–3	150	48.6	(40.0–59.0)	44.5	(35.7–55.3)	
	>3	50	38.2	(26.0–56.0)	24.3	(13.5–43.8)	
	NA	14	0.0	(63.3–100.0)	0.0	(63.3–100.0)	
All patients		473	57.3	(52.3–62.7)	52.3	(47.0–58.1)	0.000

All patients were followed carefully with a median follow-up time of 4.9 years (range: 6–151 months; cohort 1) and 5.4 years (range: 6–174 months; cohort 2). Postoperative follow-up consisted of physical examination and routine imaging investigations (chest X-ray and ultrasound examination of the abdominal cavity; chest/abdominal computed tomography examination was done for follow-up in SLN-positive or symptomatic patients). Routinely, surveillance was recommended every 3 months for the first 2 years, every 4 months in year 3, every 6 months for years 4–5, and thereafter annually.

### Pathological examination

The SLNs were evaluated by serial sectioning, and H&E staining was performed first. If this was negative, other slides were stained with immunohistochemical methods. The amount of SLN tumor burden was measured according to the Rotterdam criteria [13], which consist of the measure of the maximum diameter (in any direction) of the largest lesion on a slide (*N* = 363). All positive slides were examined, and this process of measuring the largest lesion was repeated. The largest value overall (which is the largest diameter measured anywhere on one slide in one patient) has been defined as the amount of SLN tumor burden (in mm). If a patient had multiple positive SLNs, the largest maximum diameter of any of the SLNs was the largest overall and thus the amount of SN tumor burden for this patient. Categories were made for SLN tumor burden as follows: <0.1 (sub-micrometastases), 0.1–1.0, and >1.0 mm. The location of the metastases was also recorded, according to the Dewar criteria for the microanatomic location of the metastasis [14] (*N* = 347). This was either subcapsular, parenchymal, combined, multifocal, or extensive.

### Statistical analyses

The statistical analysis was done using R 3.0.1 statistical software (R Core Team (2013). R: A language and environment for statistical computing. R Foundation for Statistical Computing, Vienna, Austria. URL http://www.R-project.org/). Packages: survival (Therneau T (2013). “A Package for Survival Analysis in S”. R package version 2.37-4, URL: http://CRAN.R-project.org/package=survival).

Logistic regression and survival analysis methods were used in the analysis. Potential risk factors of positive SLN (cohort 1) and metastases to non-sentinel lymph nodes (NSLN) (cohort 2) were investigated using univariate and multivariate logistic regression model. Variables with *p* < 0.10 were included in the initial stage of multivariate model building. Backward variable selection was then used to construct the final model.

The Kaplan-Meier estimates and Cox regression model were used in survival analysis. Patient’s survival was described using 5- and 8-year survival probability (with 95 % confidence interval) and survival curve plots. Overall survival (OS) time for the assessment of the prognostic value of clinical and pathological parameters was calculated from the date of primary tumor excision to the date of the most recent follow-up (censored data) or death. Clinical and pathological parameters are as follows: gender, primary tumor Breslow thickness (≤1.0 vs. 1.01–2.0 vs. 2.01–4.0 vs. >4.0 mm), presence of ulceration of primary lesion, primary tumor level of invasion according to Clark (II, III, IV, V), primary tumor pathological type (nodular melanoma [NM], superficial spreading melanoma [SSM], acral lentiginous melanoma [ALM], lentigo malignant melanoma [LMM], others), mitotic index of primary tumor (<1/mm^2^, 1/mm^2^, 2–5/mm^2^, >5/mm^2^; cohort 1 only), and presence of metastases to SLN (cohort 1 only), and additionally in cohort 2: localization of lymphadenectomy (inguinal vs. axillary), number of lymph nodes with metastases (1 vs. 2–3 vs. ≥4), presence of extracapsular invasion in involved lymph nodes, presence of metastases to NSLN (assessed after CLND), size of metastases to SLN according to the Rotterdam criteria, and microanatomic location of the metastasis in SLN (subcapsular, combined, parenchymal, multifocal, extensive) were tested as a factors affecting patients survival.

The multivariate Cox model was used to identify independent risk factors affecting patients’ survival. Procedure of final model building was the same as in case logistic regression model. The differences were considered statistically significant if the *p* values were <0.05.

## Results

### Survival analysis (from the date of primary tumor excision) in all patients undergoing SLN biopsy (cohort 1)

The patient characteristics and the results of an univariate analysis of the impact of individual factors on overall survival are shown in (Table 1). The median Breslow thickness of the entire group was 2.3 mm. In the analyzed subgroup of 805 patients with known mitotic index of primary tumor, we have observed the high correlation between increasing Breslow thickness and higher mitotic rate (Spearman’s correlation coefficient 0.425, *p* value <0.001).

The median 5-year and 8-year OS rates were 79.5 % (95 % confidence interval [CI]: 77.2–81.8 %) and 73.5 % (95 % CI: 70.5–76.7 %), respectively, in the entire group of patients who underwent SLN biopsy.

**Fig. 1:**
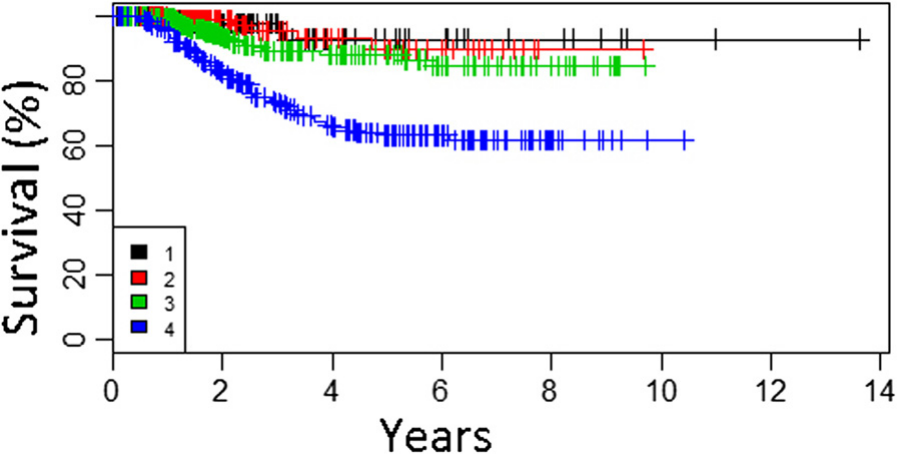
Overall survival curves according to mitotic index of primary tumor in all patients undergoing SLNB (cohort 1) (*1*, <1/mm^2^; *2*, 1/mm^2^; *3*, 2–5/mm^2^; *4.* >5/mm^2^)

**Fig. 2:**
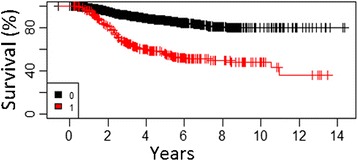
Overall survival curves according the presence of metastases to SLN (*0*—no metastases, *1*—metastases to SLN) in all patients undergoing SLN biopsy (cohort 2)

According to the multivariate analysis, we have identified three of the most important factors negatively influencing OS: mitotic index >5/mm^2^ (hazard ratio [HR] = 1.1) (Fig. 1), presence of ulceration of primary tumor (HR = 4.1), and the presence of metastases to SLN (HR = 2.2) (Fig. 2) (Table 2).

### Factors influencing on the presence metastases to SLN

In 351 cases (19.9 %), we found positive SLNs (347 of them underwent completion lymph node dissection [CLND]). Based on the univariate logistic regression model, we have found the following factors related to presence of metastases to SLN: male gender (*p* = 0.002), Clark level >II (*p* < 0.01), presence of ulceration of primary tumor (*p* < 0.001), and Breslow thickness of primary tumor >2 mm (*p* < 0.001). In multivariate analysis (Table 3), male gender Clark level IV or V and ulceration of the primary tumor were independently related to the presence of metastases to SLN.

**Table 3:** Multivariate analysis for overall survival in patients undergoing SLN biopsy (cohort 1)

Parameter	HR	95 % CI	*p* value
Mitotic index	1.071	1.01–1.136	0.021
Ulceration	4.114	1.766–9.585	0.001
Presence of metastases in SLN	2.184	1.125–4.24	0.021

**Fig 3:**
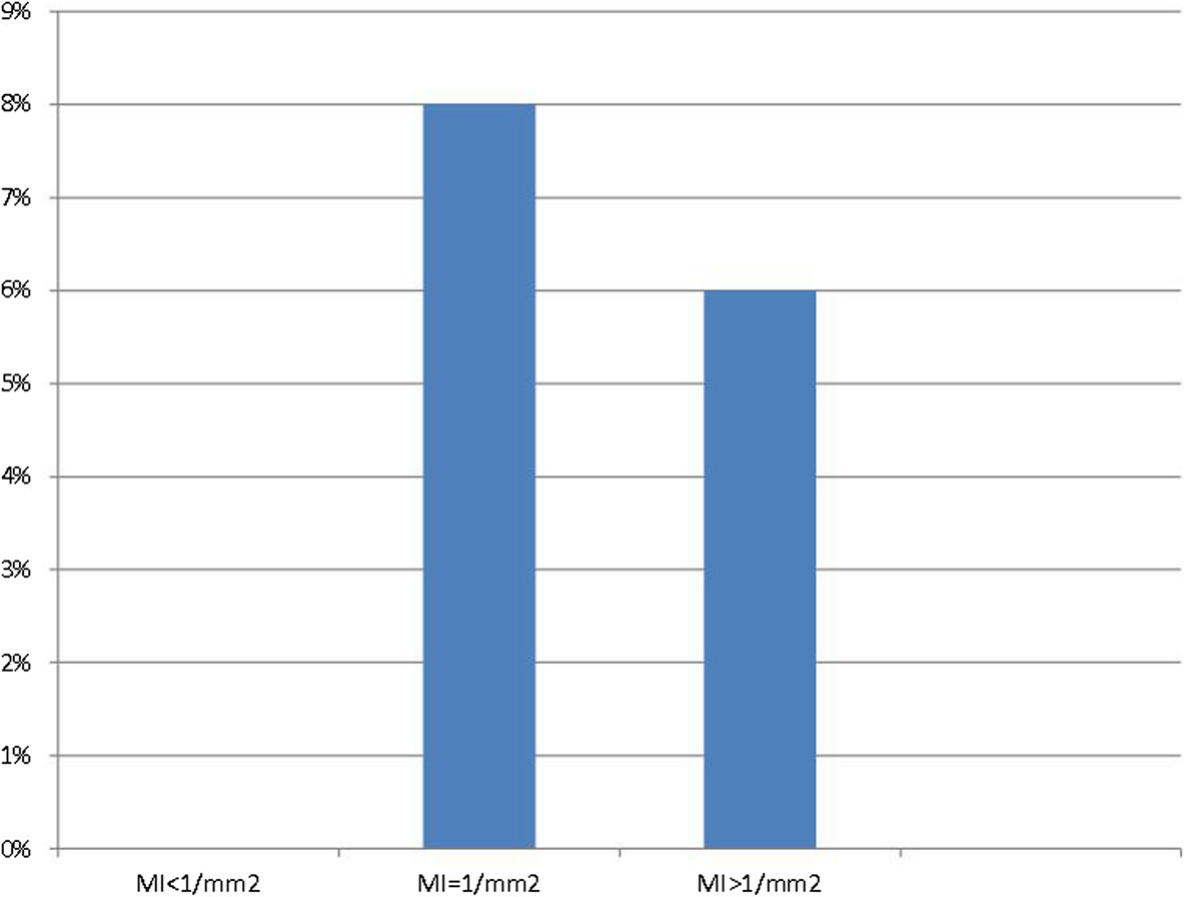
Relationship between mitotic index of primary tumor and SLN positivity in pT1 group

Moreover, in pT1 tumors (Breslow thickness ≤1.00 mm) with known mitotic index (*n* = 139), we have not found any metastases to SLNs in cases with MI < 1/mm^2^ (0/40); for 46 tumors with MI = 1/mm^2^, we detected four SLNs+ (8.7 %); and for 53 tumors with MI > 1/mm^2^, we found metastases in three SLNs (6 %) (Fig. 3).

The SLN biopsy failure rate (defined as nodal recurrences in the biopsied regional basin without simultaneous or previous local/in-transit recurrences) was 6.3 % (90/1413 SLN negative or calculated as false-negative rate 20 % 90/351 + 90). Median time to nodal relapses after false-negative SLN biopsy was 16 months.

### Analysis of factors influencing on outcomes in CLND group /cohort 2/

**Fig. 4:**
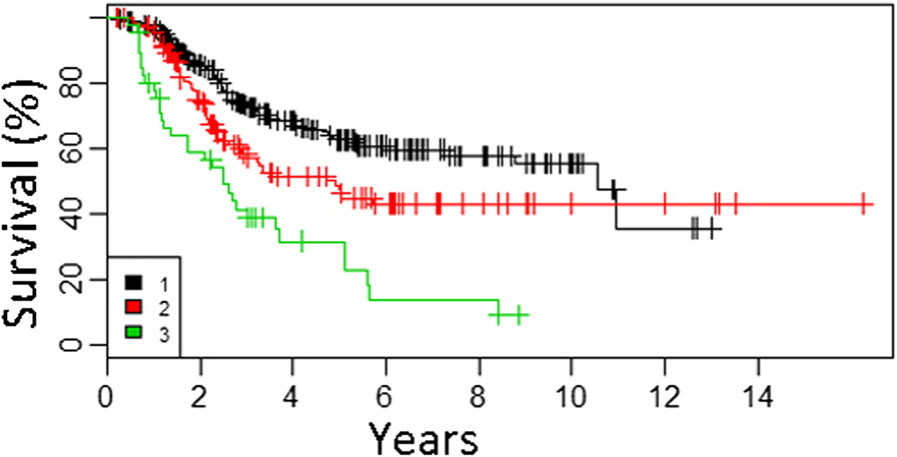
Overall survival curves according to number of lymph node with metastases in group of patients with positive SLN biopsy after CLND (cohort 2) (1–1, 2–2–3, 3 > 3)

**Fig. 5:**
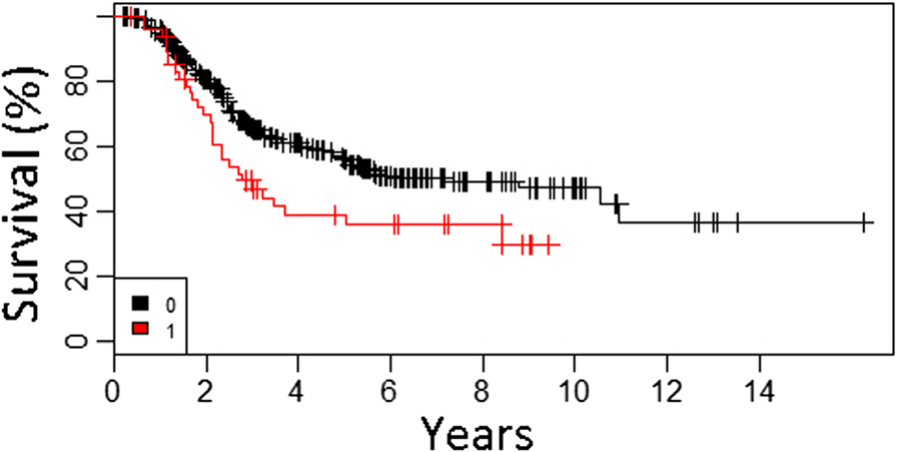
Overall survival curves according to extracapsular extension of nodal metastases (*0*—no extracapsular extension, *1*—extracapsular extension) in group of patients with positive SLN biopsy after CLND (cohort 2)

**Fig. 6:**
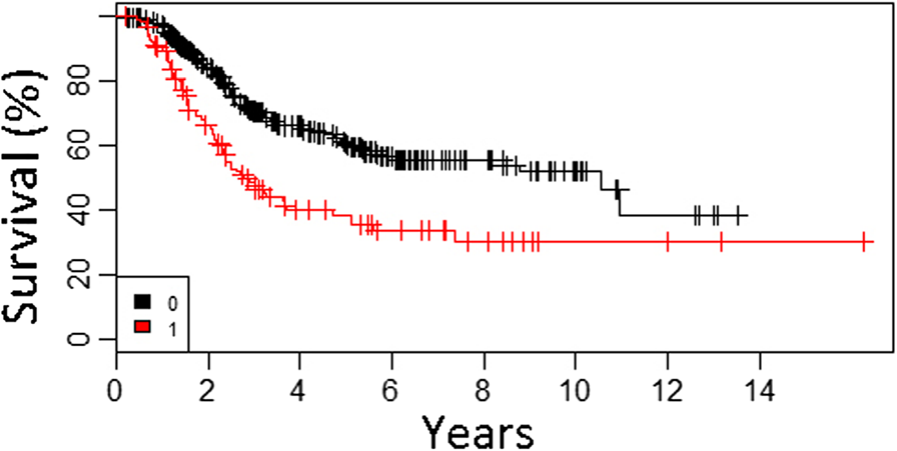
Overall survival curves according to the presence of metastases to non-sentinel lymph nodes (*0*—metastases to SLN only, *1*—metastases to SLN and NSLN) in group of patients with positive SLN biopsy after CLND (cohort 2)

**Fig. 7:**
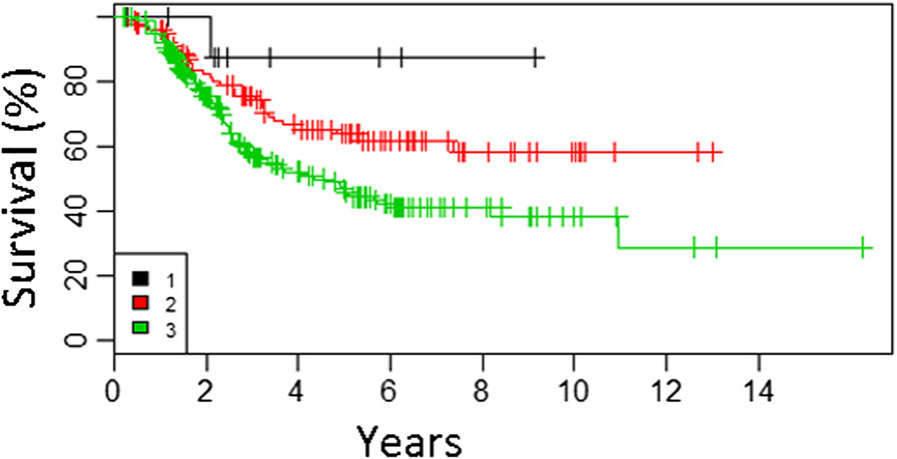
Overall survival curves according to the size of metastases to SLN according to Rotterdam criteria (SLN tumor size: *1*, <0.1 mm; *2*, 0.1–1.0 mm; *3*, >1.0 mm) in group of patients with positive SLN biopsy after CLND (cohort 2)

The patient characteristics and factors in relation to 5-year and 8-year OS rates are shown in Table 4. According to the univariate analysis, the following factors had a negative impact on the overall survival of patients after CLND: male gender, ALM primary tumor type, higher primary tumor Breslow thickness (>2 mm), ulceration of primary tumor, number of lymph node with metastases >1 (Fig. 4), extracapsular extension of nodal metastases (Fig. 5), the presence of metastases to non-sentinel lymph nodes (NSLN) (Fig. 6), the size of metastases to SLN according to Rotterdam criteria ≥1.0 mm (Fig. 7), and with the borderline significance other than subcapsular microanatomic location of the metastasis to SLN.

**Table 4:** Multivariate model—factors related to metastases in sentinel nodes

Variable	OR	ORL	ORU	*p* value
Sex (male)	1.392	1.075	1.801	0.012
Clark III	1.635	0.986	2.711	0.056
Clark IV	3.440	2.067	5.725	0.000
Clark V	6.034	3.262	11.161	0.000
Ulceration of primary tumor	2.586	1.981	3.376	0.000

According to the multivariate analysis, we have confirmed and identified that in the CLND group the most important factors negatively influencing OS are as follows: features of primary tumor (higher Breslow thickness >2.0 mm [HR = 1.01], presence of ulceration [HR = 2.5], and ALM tumor type [*p* = 4.8]) and features of nodal metastases (number of involved nodes >1 [HR = 2.05]) (Table 5).

**Table 5:** Multivariate model—factors related to metastases to non-sentinel nodes

Zmienna	OR	ORL	ORU	*p* value
Extracapsular metastases	2.022	0.939	4.352	0.072
Breslow thickness	1.102	1.042	1.166	0.001
Maximal diameter of metastatic deposit	1.068	1.013	1.126	0.015

### Factors influencing on the presence of metastases to non-sentinel lymph node (NSLN)

In 132 of 473 patients (27.9 %), we have identified additional metastases in NSLSN afer CLND.

The presence of metastases to NSLN correlated according to univariate and multivariate analyses (Table 6) with extracapsular metastases to SLN (*p* < 0.001), primary tumor Breslow thickness >2 mm (*p* < 0.001), and maximal diameter of metastases to SLN according to Rotterdam analyzed as a continuous variable (*p* < 0.001).

**Table 6:** Multivariate Cox regression model for overall survival in patients with positive sentinel lymph nodes undergoing completion lymph node dissection (cohort 2)

Zmienna	HR	95 % P.U.	*p*
Type: 2 SSM	1.214	0.778–1.892	0.393
Type: 3 ALM	4.858	2.545–9.273	0
Type: 4 LMM	1.824	0.84–3.961	0.129
Type: 5 other	0	0–Inf	0.995
Ulceration: 1	2.532	1.645–3.896	0
Breslow	1.014	1.005–1.023	0.002
Number of lymph nodes involved >1	2.05	1.453–2.893	0

## Discussion

We have confirmed the importance of SLN biopsy as a tool for accurate staging and prognosis assessment in the routine practice of patients with cutaneous melanoma. The presence of metastases to SLN found in up to 20 % of patients [8, 15] is related to almost twice less survival after 8 years from primary tumor excision as compared to patients with SLN-negative tumors. The randomized study MSLT-1 assessing prospectively the value of SLN biopsy found SLN status, Breslow thickness, ulceration presence, and localization of the primary tumor on the trunk as the independent prognostic factors related to death from melanoma in intermediate-thickness tumors [8]. We also found SLN status and ulceration of the primary tumor as independent prognostic factors for OS in the entire group of patients undergoing SLN biopsy. However, we have also demonstrated the independent negative value of high mitotic index of the primary tumor in this group of patients (which correlates with the tumor Breslow thickness). This newly introduced criterion for melanoma staging and prognosis replaced Clark’s level of invasion for thin melanomas and is thought to be related with patients’ survival [9, 16]. We also found the inverse correlation between primary tumor mitotic rate and OS, especially pronounced for a mitotic index higher than 5/mm^2^. Our results highlight the value of routine pathologic reporting of the mitotic index of primary melanoma as it is an independent prognostic factor for patients with localized primary melanomas in clinical stages I and II undergoing SLN biopsy and it is the first one-institution comprehensive study including pathological review aiming at the standardization of technique of mitotic rate assessment.

The use of SLNB reliably identifies melanoma patients with nodal micrometastases, enabling clinicians to identify patients with occult nodal metastases that would otherwise take months or years to become clinically palpable—it has been confirmed by the MSLT-1 trial data that cumulative rates of nodal involvement in patients undergoing SLN biopsy or not are similar. Currently, the positive result of SLN biopsy is a major manifestation of stage III melanomas. The results of the current study confirmed our previous data, as well as data from the AJCC staging database, demonstrating that in a group of patients with micrometastases both primary tumor features as well as nodal characteristics have independent prognostic value for OS assessment [17, 18]. Our results indicate also the heterogeneity of patients undergoing CLND due to positive SLN, which is strictly related to tumor load. The number of nodal metastases is still a very powerful independent predictor of survival among all patients with micrometastatic stage III disease, but microanatomic features of SLN metastases should also be taken into account when the patient’s prognosis is discussed.

In view of the SLN positivity in only one of five patients, some studies tried to predict SLN status in patients undergoing SLN biopsy [19, 20]. Although we did not analyze patients’ age as a prognostic factor [21] in our database due to differently used cutoff values in different databases and because we have focused on pathological features of primary tumor, we have confirmed that the Clark level of invasion and ulceration of primary tumor and additionally male gender correlated independently with SLN positivity. The results of our study underlines the position of ASCO and SSO, that in very thin melanomas up to 1 mm according to Breslow, the presence of a mitotic rate ≥1/mm^2^ can be an additional feature to propose to the patient to undergo SLN biopsy [22, 23].

Our study also highlights the fact that although the procedure of SLN biopsy may be quite accurate, it misses 20 % of positive nodes in patients with primary melanoma (this false-negative rate is consistent with other studies) [24].

The presence of metastases to NSLN is a highly negative prognostic factor for patient survival analyzed recently by other authors [25–28], and they found up to 30 % of SLN-positive cases after CLND (27 % in our series). According to our analysis, the positivity of NSLN is related to a 50 % higher chance of death after 8 years as compared to patients with metastases limited to SLN only. It may support the hypothesis that SLN may be a physiological barrier for melanoma spreading. It is one of the reasons for prediction of the necessity to perform CLND after positive SLN. After many attempts by different authors, there is still no universal approach based on morphologic criteria to not allow performing CLND in selected SLN-positive cases. We have confirmed in our group of patients that the Rotterdam criteria for assessment of tumor burden in SLN give the prognostic information, and this pathological factor is closely associated with the presence of metastases to additional NSLN after CLND, which has been suggested previously [14, 29, 30]. A combination of patients’ characteristics, primary tumor, and SLN features was proposed recently as a new scoring system [non-sentinel node risk score (N-SNORE)] for prediction of NSLN involvement [31]. This system includes two of the pathological factors found by us as independently related to the presence of NSLN metastases: maximum size of the largest tumor deposit in SLN (although with different categorization than according to Rotterdam criteria) and perinodal lymphatic invasion of SLN (described by us as extracapsular extension). We suggest also that primary a Breslow thickness >2 mm is independently related to NSLN metastases, but we did not analyze the regression of a primary tumor as Wevers et al. did [31]. We are convinced that further studies (as the Minitube trial organized by the EORTC Melanoma Group) will establish the criteria used to limit of performance of the unnecessary CLND [32], but currently, CLND is still the standard of care in every case of positive SLN biopsy.

## Conclusions

Using a large comprehensive patient cohort with long-term results, we have confirmed the crucial prognostic significance of SLN biopsy in cutaneous melanoma. We have also identified factors related to NSLN involvement, what may in the future limit indications for completion lymph node dissection in selected patients, although prospective studies are necessary. SLN biopsy currently provides more important prognostic information than can be derived from characteristics of the primary tumor only. For the patient with clinically node-negative disease, primary tumor ulceration and mitotic rate are very important factors in predicting the patient’s outcome. Nodal metastases tumor burden influences the prognosis of patients with positive SLN biopsy.

## Abbreviations

AJCC: American Joint Committee on Cancer
ALM: acral lentiginous melanoma
ASCO: American Society of Clinical Oncology
CCIO: Maria Sklodowska-Curie Memorial Cancer Centre and Institute of Oncology, Warsaw, Poland
CI: confidence interval
CLND: completion lymph node dissection
EORTC: European Organisation for Research and Treatment of Cancer
HR: hazard ratio
LMM: lentigo malignant melanoma
LND: lymph node dissection
MSLT-1: Multicenter Selective Lymphadenectomy Trial-1
NA: not available
NM: nodular melanoma
NSLN: non-sentinel lymph node
OR: odds ratio
ORL: lower range of odds ratio
ORU: upper range of odds ratio
SLN: sentinel lymph node
SSM: superficial spreading melanoma
SSO: Society of Surgical Oncology
TNM: tumor node metastasis
